# The seroprevalence of Rubella in pregnant women in Turkey: a meta-analysis research of 90988 Rubella IgM, 84398 Rubella IgG, and 522 avidity results

**DOI:** 10.4274/tjod.galenos.2018.89663

**Published:** 2019-03-27

**Authors:** Rıza Aytaç Çetinkaya, Ercan Yenilmez

**Affiliations:** 1 University of Health Sciences, Sultan Abdülhamid Han Training and Research Hospital, Clinic of Infectious Diseases and Clinical Microbiology, İstanbul, Turkey

**Keywords:** Rubella, pregnant women, meta-analysis, Turkey

## Abstract

**Objective:**

Rubella infection prevalence in pregnant women can vary from country to country, or even across regions in the same country. In this metaanalysis, the seroprevalence Rubella among pregnant women in Turkey in the last decade was evaluated.

**Materials and Methods:**

Studies conducted in Turkey between 2007 and 2017 were analyzed, and differences in seroprevalence between provinces were compared by evaluating Rubella immunoglobulin (Ig)-G, IgM, and IgG avidity results in pregnancy in this period. A data search was performed using the search terms Rubella, kızamıkçık, gebe, hamile, pregnancy, Türkiye, Turkey in Google Scholar, PubMed, Web of Science, Türk Medline, and the YÖK thesis database center.

**Results:**

A total of 26 articles associated with the seroprevalence of Rubella among pregnant women in Turkey were enrolled in the meta-analysis. As a result of an analysis of 84398 Rubella IgG, and 90988 Rubella IgM serology tests among pregnant women in 26 studies; Rubella IgG and IgM seroprevalence rates in pregnant woman in Turkey were found as 93.47% (95% CI: 91.72 to 95.03) and 0.783% (95% CI: 0.505 to 1.120), respectively. Rubella IgG low, intermediate, and high avidity rates were 4.66% (95% CI: 0.969 to 10.906), 7.51% (95% CI: 5.101 to 10.345), and 93.55% (95% CI: 82.584 to 99.311), respectively.

**Conclusion:**

The Rubella IgG seropositivity rate in Turkey among pregnant woman is high, whereas it is low for IgM. These rates may be considered as the result of successful immunization policies and practices. In a few provinces, it is necessary to revise the Rubella immunization procedures and adult vaccination strategies should be developed in order to control Rubella infections in adults, including pregnant women.

**PRECIS:** In this meta-analysis, studies conducted in Turkey between 2007 and 2017 were analyzed, and differences in seroprevalence between provinces were revealed by evaluating Rubella IgG, IgM and IgG avidity results in pregnancy in this period.

## Introduction

Rubella is a vaccine-preventable disease and is one of the most infectious viral diseases known in humans. Congenital Rubella syndrome (CRS), consisting of cardiac disorders, cataract, deafness, cleft palate, autism, and fetal death can occur when the Rubella virus vertically infects the fetus during pregnancy^(1)^. Owing to the vaccination practices in childhood and adults, the prevalence of Rubella in pregnant women decreased and Rubella has become a rare infection in many developed and some developing countries. Some countries in the Western hemisphere and Europe have eliminated Rubella and CRS^(2)^.

Immune status can be evaluated by enzyme-linked immuno-reactive techniques (enzyme immunoassay, ELISA). From these tests, immunoglobulin (Ig)-G antibody shows a previous infection or immunization by vaccines. The Rubella serum IgM test indicates an acute Rubella infection and must be confirmed by at least one of these tests: Rubella-RNA polymerase chain reaction or Rubella IgG-avidity or western-blot^(2)^.

The prevalence of Rubella infection in pregnant women can vary from country to country, and even across regions in the same country. In this meta-analysis, Rubella antibody tests in the last 11 years in pregnant women in Turkey were examined, and the differences in prevalence between provinces in our country, and between our country and other countries were compared.

## Materials and Methods

**Figure 1:**
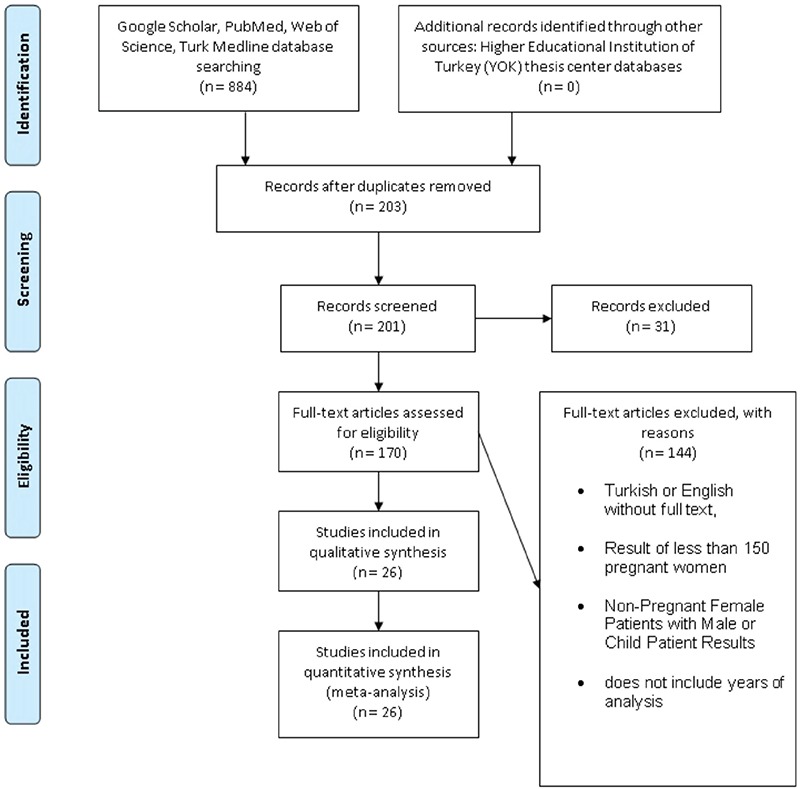
Flow chart for study selection and literature review. Summary of the literature search and study selection on Rubella antibodies in pregnant women

In this meta-analysis, studies conducted in Turkey between 2007 and 2017 were analyzed, and differences in seroprevalences between provinces were compared by evaluating Rubella IgG, IgM, and IgG avidity results in pregnancy in this period. Data in these studies were screened and evaluated using the preferred reporting items for systematic reviews and meta-analyses (PRISMA) flow-chart according to the inclusion criteria (Figure 1).

### Source of data

A data search was performed using the search terms Rubella, kızamıkçık, gebe, hamile, pregnancy, Türkiye, Turkey in Google Scholar, PubMed, Web of Science, Türk Medline, and the YÖK thesis database center by two independent researcher in 2018.

### Inclusion and exclusion criteria

Studies with Rubella IgM, IgG, and IgG avidity test results in pregnant women in Turkey between 2007 and 2017 were included in the study. Original articles with antibody test results of at least 150 pregnant women with full text in Turkish or English were recorded. Studies were excluded if they included the results of the antibodies in non-pregnant women and male or child patients, and if the studies did not include the study period or were published after 2007 but the data were collected before 2007 (Figure 1).

### Data search and collection of data

Considering the criteria, data were screened and evaluated by two different researchers (RAC, EY) in order to prevent publication bias. Study data: author’s surname, date of publication, years of tests performed, numbers (n) and rates (%) of Rubella IgG, IgM, and IgG-avidity tests, and province of the study performed were recorded in the Microsoft Office 2016 Professional Plus Excel program. Before the meta-analysis, all data were listed in alphabetical order according to the author’s surname in the extended format. Disputes between researchers were resolved by mutual discussion.

### Statistical Analysis

Medcalc© software version 17.9.7 program was used for meta-analysis. Data were transferred from the Excel program. A funnel plot was used to evaluate possible bias and the results were interpreted.

A statistical test for heterogeneity was performed to measure the data heterogeneity. According to this; I^2^≤25% heterogeneity was assumed to be insignificant and a fixed effect was used. I^2^>25% heterogeneity was assumed to be insignificant; the study data were considered as nonhomogeneous and the random effect value was used. P<0.01 was considered to be no need to add more studies.

## Results

In this meta-analysis, 884 articles were found in accordance with the research criteria (Figure 1). A total of 681 articles were excluded from the study because of repetition in two or more different databases. After the removal, we had 203 studies, 201 of which we could screen. After evaluation of the study title and summary, another 31 were excluded from the study. After full text evaluations, 144 of the 170 studies were excluded from the study according to the determined criteria. As a result, a total of 26 articles associated with Rubella seroprevalence in pregnant women in Turkey were enrolled in the meta-analysis.

The studies included in the meta-analysis were from Afyon (n=2), Artvin (n=1), Bingöl (n=1), Denizli (n=1), Edirne (n=1), Isparta (n=1), Istanbul (n=4), Kahramanmaraş (n=1), Konya (n=2), Manisa (n=1), Muğla (n=1), Middle Black Sea (n=1), Rize (n=1), Uşak (n=1), Van (n=2), Yozgat (n=1), Zonguldak (n=1).

The study of Sargın and Saygan^(3)^ from Ankara had the maximum number of cases with 31385 pregnant women, and the study of Bakacak et al.^(4)^ from Kahramanmaraş had high number of cases with 11823 pregnant women.

**Figure 2:**
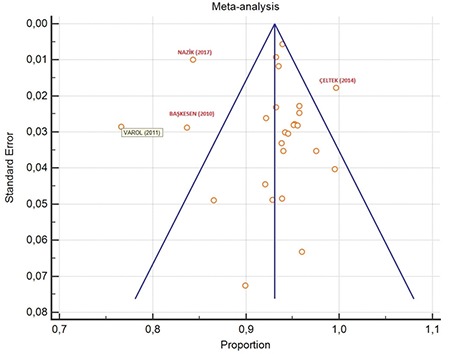
Funnel plot analysis graph of anti-Rubella immunoglobulin-G in Turkey

**Figure 3:**
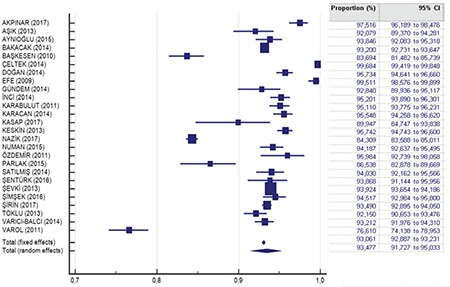
Meta-analysis graph of anti-Rubella immunoglobulin-G

According to the meta-analysis of 84398 serologic tests of pregnant women in the 26 studies, the seroprevalence rate of Rubella IgG in pregnant woman in Turkey was 93.47% (95% CI: 91.72 to 95.03). The Cochrane Q test was 2032,5378; I^2^=98.77% and p<0.0001, respectively (Table 1). In funnel plot analysis, minimal asymmetry was found in the studies of Varol et al.,^(5)^ Nazik et al.,^(6)^ Başkesen et al.,^(7)^ and Çeltek et al.^(8)^ (Figure 2,Figure 3). Overall, the asymmetry test showed no bias.

**Figure 4:**
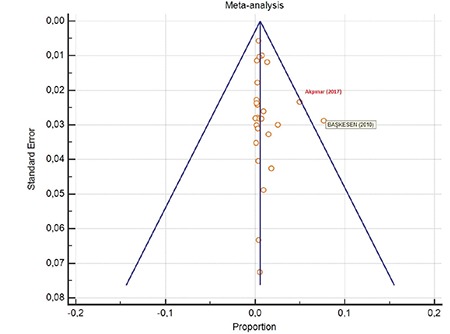
Funnel plot analysis graph of anti-Rubella immunoglobulin-M

According to a meta-analysis of 90,988 serologic tests of pregnant women in the 26 studies, the seroprevalence rate of Rubella IgM in pregnant woman in Turkey was 0.783% (95% CI: 0.505 to 1.120). The Cochrane Q test was 583,6836; I^2^=95,72% and p<0.0001 (Table 2). A negligible asymmetry was found in the funnel plot analysis, and the asymmetry test showed no bias (Figure 4). The results of Başkesen et al.^(7)^ and Akpınar et al.^(9)^ were most distant from the average value of Turkey.

Additionally, Rubella IgG-avidity rates were analyzed in the present meta-analysis (Table 3). Accordingly, the low avidity rate was 4.66% (95% CI: 0.969 to 10.906) and the Cochrane Q test was 10,3230; I^2^=70.94% (p<0.0001) in 427 pregnant in four studies^(10,11,12,13)^, intermediate avidity rate was 7.51% (95% CI: 5.101 to 10.345), and the Cochrane Q test was 1,8145; I^2^=0.00 (p=0.404) in 384 pregnant in three studies^(11,12,13)^, the high avidity rate was 93.55% (95% CI: 82.584 to 99.311), and the Cochrane Q test was 46,4845, I^2^=91.39% (p<0.0001) in 522 pregnant women in five studies^(3,10,11,12,13)^.

## Discussion

Rubella infection is usually subclinical in childhood, but may be more severe at older ages in life. It can also lead to severe anomalies or death in the fetus in the first trimester of pregnancy^(1,14)^.

The serologic test showing previous Rubella infection or immunization status alone is the Rubella IgG antibody. Due to the increase in the awareness of pregnant women and socioeconomic developments in our country, there has been an increase in prenatal screening tests in recent years. According to the meta-analysis of 26 studies analyzed, the Rubella IgG seropositivity rate in Turkey was 93.4%. This rate was higher than 44% of the studies, and lower than 56% of the studies included in the meta-analysis. The study of Şevki et al.^(3)^ in Ankara with 31,385 cases represents the Turkey’s average best; the seropositivity rate of 93.9% and 37.1% weight in the meta-analysis^(3)^.

In a general perspective, it is considered that the vaccination rates in the west part of Turkey is higher, and in the east part of Turkey, immunity is gained after acquiring infections against infections that can be prevented by vaccination. The study of Varol et al.^(5)^ conducted in the Thrace region of Turkey revealed the lowest IgG ratio (76.6%), and also Baskesen et al.^(7)^ from Manisa revealed a lower rate (83.6%) than the average of Turkey. In these two studies, it was not possible to make an inference because the age status of the pregnant women was not given in cross-sectional intervals. However, these rates raise doubts about effective vaccination strategies in the regions where both studies were conducted. More comprehensive randomized controlled prospective research is needed for these two regions.

Rubella IgG seropositivity rate was 84.3% in the study conducted by Nazik et al.^(6)^ in the Bingöl province in 10,178 pregnant women. Similarly, the study of Parlak et al.^(14)^ in the Van province in 416 pregnant women, the rate was 86.5% for IgG seropositivity. These rates could be considered to be due to the low level of vaccinations of the people living in the region, but it is not possible to form a definite opinion on this issue.

In the study of Çeltek et al.^(8)^ with 3162 pregnant women representing the Middle Black Sea region, Rubella IgG seropositivity rate was found as 99%. Çeltek et al.^(8)^ interpreted their result as close to the average of Turkey. However, the ratio in their study was found to be higher than the average of Turkey according to our meta-analysis. The high rate in this region indicates that the number of pregnant women who could be infected with Rubella during pregnancy was low. In order to use the results across the country, the characteristics of the cases should be evaluated further.

The study of Özdemir et al.^(15)^ was not included in the meta-analysis because the test and technique used in the multicenter study conducted in seven provinces were not explicitly written. In this multicenter study, Rubella IgG positivity was between 76-96.4% and the results varied significantly in different provinces. Our meta-analysis, or similarly, this multicenter study shows that different results can be obtained in different regions. This difference can be explained by the wide geographic structure of Turkey and by the sociocultural differences that exist between the regions. Therefore, we believe that accurate rates throughout the country can only be obtained by meta-analysis studies.

The age and dose of the Rubella vaccination may vary depending on a country’s vaccination policy. In general, an 84.7% seropositivity rate is achieved by single-dose vaccination, and this rate reaches 90% with two doses of vaccine (ages 1 and 5)^(16)^. In our country, Rubella vaccines are administered as two doses in children at 12 months and 7 years. Turkey, with a rate of 93.4% Rubella IgG seropositivity, has a rate just above that in the general literature. Turkey’s neighboring Iran has a seropositivity rate of 94%,^(17)^ and Greece, which is a member of the European Union, has a seropositivity rate of 97% in women with childbearing age^(18)^. This ratio is 89.3% in Brazil and 99.3% in the United States of America^(16)^. In India, which is a Far East country, it is 68.3%. The reason for this low rate is that Rubella vaccine is currently not included in the national immunization program^(19)^. In China, although this rate varies according to age in pregnant women, this rate is approximately 84.0%^(20)^. The high level of IgG antibody seropositivity in our country reveals that we have a successful vaccination policy compared with other developing countries.

It is reported that if immunity against Rubella is below 90%, the risk of acute infection and CRS might increase in childbearing age^(9)^. Rubella IgM antibody is the first-step test in determining acute infection. In our meta-analysis, the IgM seroprevalence rate in Turkey was about 0.8%. The study of Başkesen et al.,^(7)^ which included 1202 pregnant women in the Manisa region, is one of the most important studies on this subject with 7.6% IgM seropesitivity rate and 1.3% weight in meta-analysis. In patients in Manisa, clinical evaluations should be made, IgM results, cross-reactions or primary/re-infection should be confirmed, and it should be kept in mind that antibody positivity may persist for a year following vaccination or asymptomatic infection. There is a need for a prospective randomized controlled trial in the Manisa region to identify these patients.

As a part of the “Elimination of Rubella and CRS Prevention Program”, Rubella vaccine has administered in Turkey, including Isparta, since 2006^(21)^. The Rubella IgM seropositivity rate was found as 4.9% in the retrospective study of Akpınar et al.^(9)^ in Isparta, which comprised 1829 pregnant women. There is not enough information about whether pregnant women are vaccinated because of the fact that the study was retrospective; however, it can still be concluded that the routine vaccination program is not adequately successful in the Isparta province.

In pregnant woman with both IgG and IgM positivity, an IgG avidity test should be performed to estimate the time of infection. According to our analysis, Rubella IgG avidity was studied in only 5 studies in Turkey, and the low avidity rate was 4.6% in a total of 427 pregnant women. One of the reasons for this high rate was the study of Şimsek et al.^(11)^ conducted in Afyon. In this study, there were 28 patients with IgM positivity and 44 patients with gray-zone IgM positivity, but avidity was studied in only 12 of them. Two patients with low avidity in this group increased the rate of high avidity in the analysis.

It is not possible to make a detailed and direct comparison through avidity results because publications about CRS are generally case studies in our country. There is a need for a multicenter prospective study for our country including CRS patient series with validation tests, advanced diagnostic tests, and large-scale IgG-avidity results.

There are some restrictions for meta-analyses; there may be studies that have not completed the publication procedure in the analysis period and also there may be publications for which the full text cannot be reached. There may also be changes in the interpretation of the study results due to differences in the kit and devices used in the studies analyzed.

In summary, our study cannot show the whole the Rubella seroprevalence in Turkey. The high seroprevalence of IgG antibodies in Turkey may be considered as the result of successful immunization policies and practices. In a few provinces, it is necessary to revise the Rubella immunization procedures and adult vaccination strategies should be developed in order to control Rubella infections in adults including pregnant woman.

## Figures and Tables

**Table 1:**
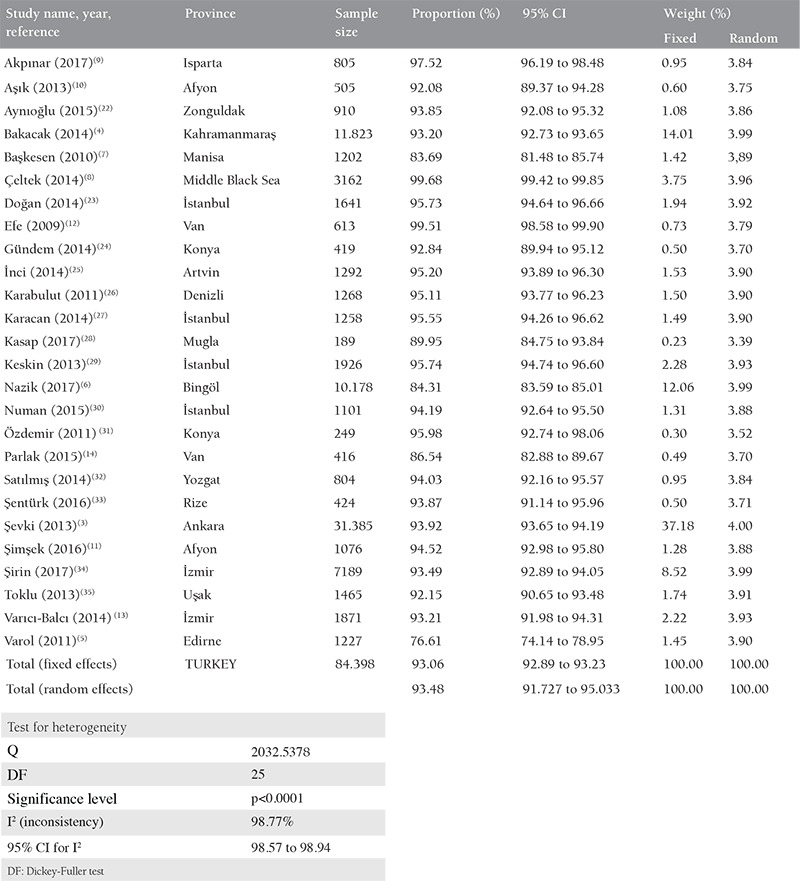
Meta-analysis of anti-Rubella IgG among pregnant women in Turkey

**Table 2:**
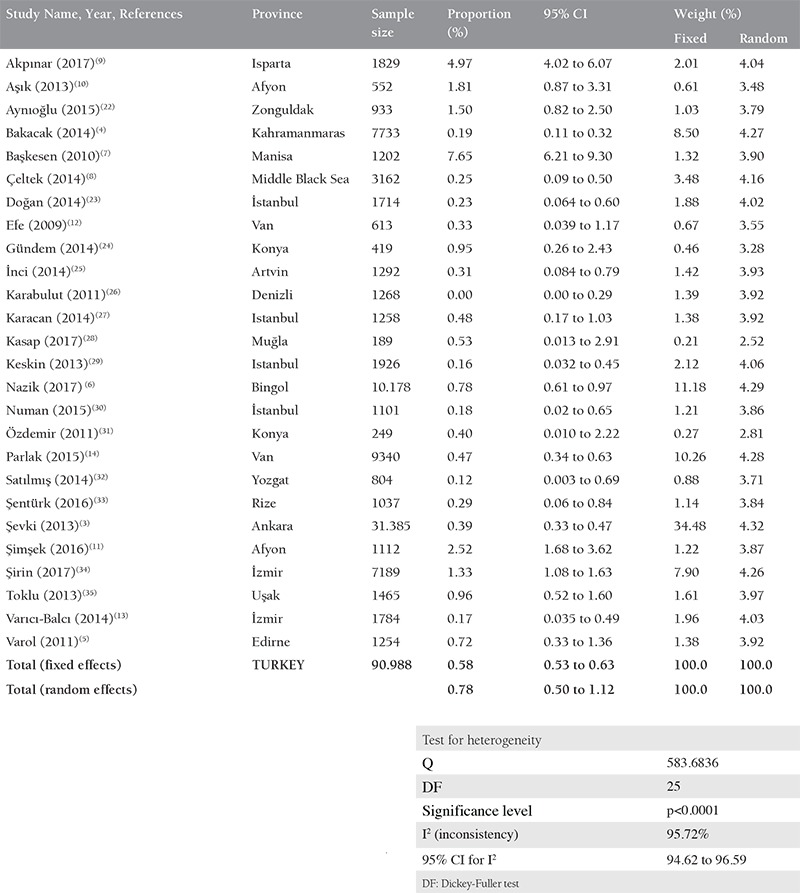
Meta-analysis of anti-Rubella immunoglobulin-M among pregnant women in Turkey

**Table 3:**
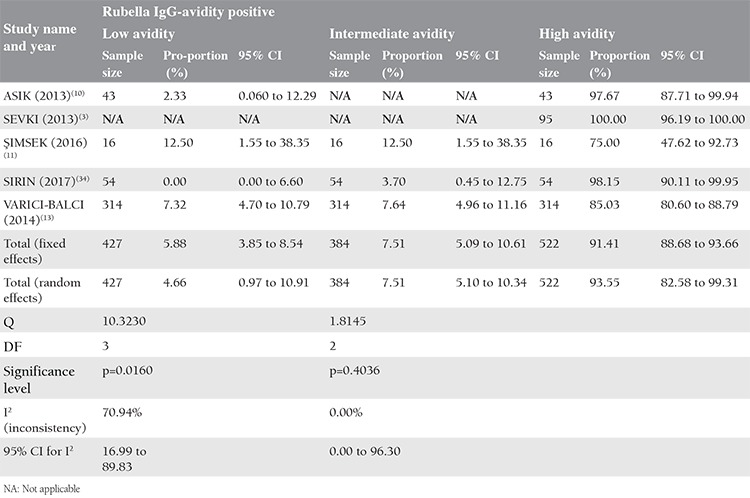
Meta-analysis of Rubella immunoglobulin-G-avidity test results
